# Food Defense Risk Assessment of an Edible-Coating Processing Line: A Comparative Case Study Using TACCP and CARVER+SHOCK

**DOI:** 10.3390/foods15172997

**Published:** 2026-08-26

**Authors:** Kharla Andreina Segovia-Bravo, Cristina Campanero Pintado, Efrén Pérez-Santín

**Affiliations:** Escuela Superior de Ingeniería y Tecnología (ESIT), Universidad Internacional de La Rioja (UNIR), Avenida de la Paz, 137, 26006 Logroño, Spain

**Keywords:** food defense, edible coatings, citrus processing, TACCP, CARVER+SHOCK, risk assessment

## Abstract

The U.S. FSMA Intentional Adulteration (IA) Rule and GFSI-recognized certification schemes such as IFS Food and BRCGS Food include requirements for establishing and maintaining a written food defense plan, including vulnerability assessment. This study evaluates the suitability and policy relevance of two established food defense risk assessment methodologies, TACCP and CARVER+SHOCK, when applied to the processing of whole oranges with edible coatings. A structured case study was conducted at a medium-sized citrus processing facility certified under IFS Food and BRCGS Food standards. Food defense threats were systematically identified along the entire production line using the FDA Food Defense Self-Assessment Tool. The identified threats were subsequently assessed using TACCP, which is based on likelihood and impact, and CARVER+SHOCK, a multidimensional quantitative approach integrating operational, economic, and psychological impact factors. Twenty-four potential intentional contamination threats were identified. Both methodologies consistently identified critical vulnerabilities at key control points, particularly in washing systems, chemical handling areas, and stages with unrestricted access. TACCP proved to be rapid, intuitive, and easily integrated into existing food safety management systems. In contrast, CARVER+SHOCK provided a more differentiated ranking of the identified threats because of its multidimensional scoring structure, thereby supporting the development of a structured and economically rational mitigation plan and offering value for policymakers and certification bodies seeking to harmonize risk-assessment practices.

## 1. Introduction

Food defense has become a critical concern within the broader field of food safety, driven by increasingly complex supply chains and heightened consumer expectations regarding safety and authenticity. It is also included as an auditable requirement in all GFSI-recognized standards, including IFS Food and BRCGS Food [1,2]. In regulatory practice, the U.S. FSMA IA Rule [3] formalizes these expectations by mandating a written food defense plan that covers vulnerability assessment, mitigation, monitoring, corrective actions, verification and training. The rule explicitly considers the ‘inside attacker’ scenario.

Food defense focuses on protecting food products from intentional contamination or adulteration intended to cause public health harm or economic disruption [4,5]. It addresses ideologically or behaviourally driven threats, including sabotage, terrorism, or criminal tampering. Food defense should be distinguished from other related concepts. Food fraud generally refers to economically motivated deception intended to obtain financial gain and is commonly addressed through vulnerability assessment systems such as VACCP [6]. In contrast, food defense focuses on intentional malicious acts designed to cause public health, economic, or societal harm. Such acts may include sabotage, intentional adulteration or, in extreme cases, terrorism-related actions targeting food systems [7,8]. The present study focuses exclusively on food defense threats and does not evaluate food fraud vulnerabilities.

As the food system becomes more interconnected and reliant on global networks, the potential for intentional adulteration increases, necessitating robust risk mitigation strategies [9]. In this scenario, effectively managing food defense threats requires a structured risk analysis framework comprising three key stages: (1) identifying potential food defense threats; (2) assessing the risk; and (3) implementing control measures to mitigate the risks [10]. This three-step approach is not only central to HACCP systems but also underpins specialized methodologies such as VACCP (Vulnerability Assessment and Critical Control Points) and TACCP (Threat Assessment and Critical Control Points), which address economically motivated adulteration and intentional contamination, respectively [6].

The first step, threat identification, is typically well supported by the scientific literature, technical guidelines and the operational realities of the companies and their facilities, especially for conventional food products. Likewise, the third step, risk management, is generally straightforward once vulnerabilities and threats are identified, as industry standards provide clear recommendations for mitigation strategies. The intermediate step, risk assessment, remains a complex and often ambiguous task, especially in the context of emerging food technologies such as edible-coated whole oranges. A major challenge lies in the absence of standardized and mandatory protocols for selecting and applying risk matrices tailored to food defense scenarios. Current regulatory and voluntary frameworks provide some guidance [11,12], but their approaches differ and can create confusion within the industry because they do not mandate the use of specific matrix models for particular food processes. This lack of clear guidance means that the selection of a risk matrix is frequently left to the discretion of the assessor, whose decisions may be influenced by personal experience, subjective judgment, or organizational preferences. As a result, the consistency and reliability of risk evaluations can vary significantly across different assessments.

Innovative food products, such as whole fruits with edible coatings, introduce new challenges in this landscape. These coatings are designed to extend shelf life and enhance appearance [13]. However, it is important to distinguish between vulnerabilities that are already present in conventional citrus-processing operations and those associated with edible coating technology itself. Conventional facilities commonly face food defense challenges related to raw material reception, washing systems, storage, transportation, and traceability controls. In contrast, edible coating operations introduce additional ingredients, preparation stages, dipping systems, draining procedures, drying operations, and associated handling activities, creating additional vulnerability nodes that must be considered within food defense assessments [14]. For example, the immersion tank used to apply final coating solution and ambient temperature drying chambers can represent sources of vulnerability because of their open design and prolonged exposure times [15]. These steps are not commonly found in traditional citrus packing lines and therefore require dedicated food defense risk assessment approaches. The open design of these processes can facilitate intentional contamination. Specifically, the product could be subjected to a malicious biological or chemical attack during the coating application stage, and the water tanks and conduits could likewise be contaminated with such agents [4,5].

Oranges rank among the most extensively cultivated and commercially traded fruits worldwide, constituting over 50% of global citrus production and more than 40% of citrus exports [16]. Their widespread consumption is largely attributed to their nutritional benefits, particularly their high content of vitamin C. Data from the USDA Foreign Agricultural Service indicate that global orange production has remained relatively stable in recent years, averaging between 47 and 50 million metric tons annually. Major producing countries include Brazil, the United States, China, and India, with Brazil alone contributing approximately 30% of the global output [17]. From a trade perspective, the international export value of oranges reached an estimated $5.13 billion in 2024. The leading exporters are Spain, South Africa, Egypt, The United States, and The Netherlands, and together account for over 67% of global orange exports [18].

Within this dynamic global market, the use of edible coatings on fresh oranges and other fruits has garnered increasing attention due to their ability to prolong shelf life, minimize postharvest losses, and enhance food safety. These coatings are generally formulated from natural biopolymers, such as polysaccharides, proteins, and lipids, and applied as thin layers to the fruit surface. They function as semi-permeable barriers that regulate gas exchange, reduce moisture loss, and inhibit microbial growth [19]. Additionally, the incorporation of active compounds such as essential oils, organic acids, and antioxidants into the coating matrix further enhances their antimicrobial and antioxidant efficacy, contributing to improved safety of the final product [14].

This study specifically focuses on a comparative evaluation of the CARVER+SHOCK and TACCP assessment methodologies as applied to the processing of whole oranges with edible coatings for food defense purposes. Despite the widespread use of TACCP and CARVER+SHOCK in food defense programs, limited comparative evidence is available on how these methodologies perform when applied to emerging fresh-produce systems that incorporate novel postharvest technologies. Edible coating operations introduce additional ingredients, preparation stages, dipping procedures, drying steps, and extended product exposure periods that may influence the identification and prioritization of food defense vulnerabilities.

Consequently, the scientific interest of this study lies not in developing a new food defense assessment methodology but in evaluating how two established approaches perform when applied to the same industrial context. Understanding the differences in vulnerability prioritization, risk characterization, and mitigation planning produced by TACCP and CARVER+SHOCK may help practitioners select the most appropriate assessment framework according to the characteristics of their operation.

Therefore, this study compares the application of TACCP and CARVER+SHOCK within a citrus-processing facility producing edible-coated oranges. The objective is to analyse their respective prioritization outcomes, practical applicability, and implications for food defense mitigation planning in this emerging processing context. By providing case-specific comparative evidence, the study contributes to the discussion on the applicability and potential harmonization of food defense risk assessment approaches used in industrial practice.

## 2. Materials and Methods

### 2.1. Case Study Description

A case study design was selected because the objective of the research was to perform an in-depth evaluation of food defense vulnerabilities within a specific industrial process incorporating edible coating technology. This approach allows a detailed examination of operational characteristics, process-specific vulnerabilities, and mitigation planning considerations that cannot be adequately captured through generalized datasets or secondary data sources. The case study therefore serves as a framework for comparing the practical application of food defense assessment methodologies under real industrial conditions.

*Company profile:* The case-study company is a mid-sized agro-industrial company located in Valencia, Spain, specializing in the processing and export of fresh oranges. The company is certified under IFS Food and BRCGS Food standards and operates within the value-added fresh produce sector, focusing on minimally processed fruits. The facility comprises a 4500 m^2^ processing plant with a production capacity of approximately 12,000 tons of oranges per year and 75 employees. Its products are distributed both domestically and internationally, with key export markets in Germany, France, and the Netherlands.

In terms of food defense, the facility has implemented documented food defense training programmes for employees, restricted-access policies, visitor registration procedures, supervision systems, and external physical security measures. These controls were considered during the threat identification stage; however, their presence does not eliminate residual vulnerabilities or insider threats, which remain inherent considerations in food defense assessments.

In response to evolving market demands and technological advancements, the company has recently initiated the production of whole oranges coated with edible films. This innovation aims to extend the product’s shelf life and enhance its long-term safety profile. Consequently, to ensure full compliance with IFS Food and BRCGS Food standards, it is imperative for the company to perform a comprehensive food defense threat analysis of the updated production process and to redesign its food defense plans accordingly.

Although the present study focuses on a single industrial facility, the selected company reflects several characteristics commonly found in medium-sized citrus-processing enterprises operating within the European fresh-produce sector, including certification under GFSI-recognized standards, export-oriented operations, conventional citrus-processing activities, and the implementation of edible coating technology. Nevertheless, the study was not designed to provide statistically representative conclusions for the entire citrus industry, as food defense vulnerabilities and mitigation strategies are inherently site-specific and depend on the operational, organizational, and infrastructural characteristics of each processing facility.

### 2.2. Raw Materials

*Oranges:* The oranges are of the *Navelina* variety and medium size (180–220 g per unit).

*Plastic boxes:* Plastic boxes used for packaging are made of high-density polyethylene (HDPE) or polypropylene (PP), materials known for their mechanical strength, chemical resistance, and compliance with food-contact regulations. These boxes are designed to be stackable, ventilated to prevent moisture accumulation, and easy to sanitize.

*Labels:* Labels are received in protected rolls, with an external sample displayed for visual validation. They are printed using food-grade inks and adhesives and are designed to withstand humidity and temperature variations during storage and transport. The label substrate is often made of paper with a protective plastic film coating to ensure durability and legibility.

Pallets: The pallets are received in standardized format (EUR-pallet) with dimensions of 1200 mm × 1000 mm. They are made of heat-treated (HT) wood and meet ISPM 15 requirements.

*Disinfectants and fungicides:* Imazalil is received in emulsifiable concentrate (EC) form, with a concentration of 500 g/L of active ingredient. Imazalil is an authorized post-harvest fungicide commonly used in citrus processing within the European Union. Compliance with the applicable maximum residue levels (MRLs) established under Regulation (EC) No. 396/2005 [20] and its subsequent amendments constitutes an important food safety and food defense requirement. Since intentional misuse, substitution, adulteration, or overdosing of fungicides could create significant chemical hazards, verification of supplier specifications, application conditions, and regulatory compliance are essential control measures. At the time of writing, the EU MRL established for imazalil in citrus fruits is 7 mg/kg [21]. During the drenching treatment, the commercial imazalil formulation (500 g/L) was diluted to obtain a working concentration of 300 mg/L. This concentration is consistent with authorized post-harvest application conditions for citrus fruits and was applied in accordance with the manufacturer’s instructions and the company’s approved operating procedures to ensure compliance with the applicable EU maximum residue limits (MRLs) in the final product.

Sodium hypochlorite is received in liquid solution form at industrial concentrations of 12–15% available chlorine (trade percent), equivalent to 120–150 g/L. Because imazalil and sodium hypochlorite are directly applied during processing and may encounter products intended for human consumption, their handling is relevant not only from a food safety perspective but also from a food defense perspective. Intentional misuse, substitution, adulteration, or overdosing of these substances could result in significant chemical hazards. Therefore, supplier approval, verification of technical specifications, secure storage conditions, compliance with approved application requirements, and adherence to applicable maximum residue limits constitute important control measures within both food safety and food defense programmes [22,23,24].

*Edible coating ingredients:* Acetic acid is received in aqueous solution form at concentrations of 10–30% *w*/*v*. It is food-grade and accompanied by a technical data sheet (TDS) and certificate of analysis (COA) to ensure compliance with purity and safety standards. Rosemary extract is received in liquid form, standardized to contain 5–33% of carnosic acid and carnosol. Glycerol is received as a viscous, colourless liquid with a purity of ≥99.5%, and is fully miscible with water. It is food-grade and compliant with European food additive regulations (E422). Chitosan is received in powdered form with a degree of deacetylation of 75–90%.

### 2.3. Process Flow Description

**Figure 1:**
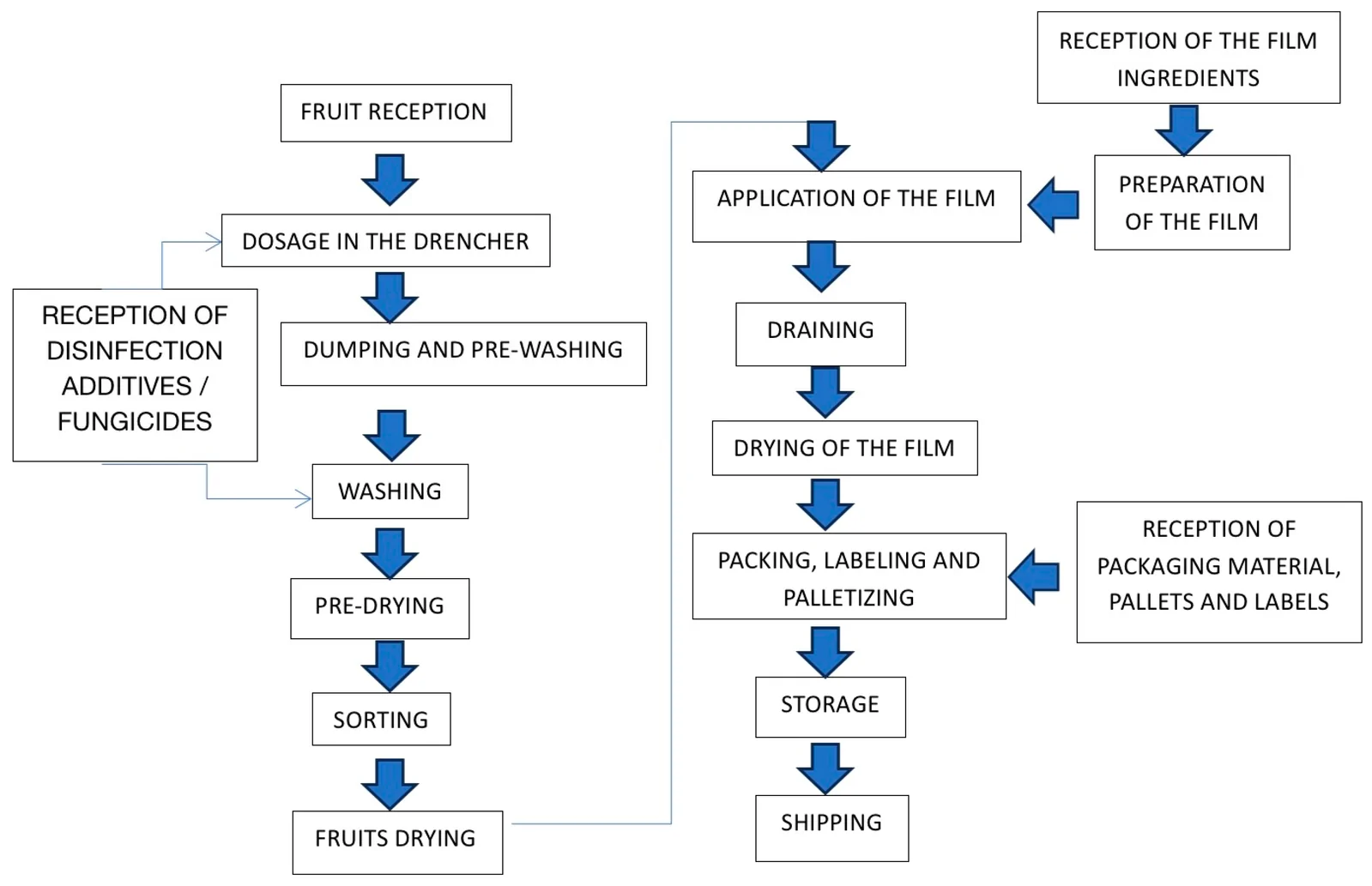
Process flow chart of the production of edible-coated oranges.

Figure 1 shows the full production process for coating whole oranges with edible films, from raw material reception to refrigerated distribution. The diagram provides an integrated overview of all processing stages.

**Raw material reception**. During the reception stage, it is essential to identify the product, unload it while adhering to good handling practices, and conduct an inspection control to ensure that the received citrus fruits meet the requirements agreed upon with the supplier regarding safety, quality, legality, and authenticity. To achieve this, a thorough document review is essential to confirm compliance with the supplier approval plan. One requirement for the orange supplier is GLOBAL G.A.P. certification.

**Reception of packaging, packing, and labelling materials**. Oranges are packaged in plastic boxes, and labels are received on protected rolls, which display a sample of the label on the exterior for validation. The supplier approval plan includes controls for the safety and quality of food-contact materials and requires compliance with applicable legal requirements concerning good manufacturing practices and the declaration of conformity of food-contact materials.

**Reception of disinfectants and fungicides**. The fungicide imazalil is received in emulsifiable concentrate (EC) form. The technical data sheet and product analysis report are verified to ensure compliance with the requirements agreed upon with the supplier regarding legality and quality [20,21]. Sodium hypochlorite is received in solution form. The technical data sheet and product analysis report are also verified to ensure compliance with the agreed-upon requirements with the supplier regarding legality and quality.

**Reception of film ingredients.** Acetic acid is received in solution form, along with a rosemary extract solution, glycerol, and powdered chitosan. The technical data sheet and product analysis reports are verified to ensure compliance with the requirements agreed upon with the suppliers regarding legality and quality.

**Drencher treatment.** After unloading, the oranges are subjected to a shower of water containing the fungicide imazalil to protect the fruit from fungal attack that may cause deterioration. To ensure the safety of the oranges, the applicable maximum residue limits (MRLs) are observed.

**Dumping and pre-washing**. The oranges are dumped into a basin of water to cushion the impact of the fruit falling onto a rigid surface. This process also removes soil particles, leaves, other field debris, and excess fungicide from the fruit. The oranges are removed from the basin using conveyor belts.

**Disinfection with sodium hypochlorite**. The conveyor belts transport the fruit to a second tank where the oranges are washed more thoroughly in water containing sodium hypochlorite as a disinfectant. The washing water parameters, including pH, free chlorine concentration, water temperature, and exposure time to the disinfectant, are monitored.

**Rinsing**. The oranges are removed from the tank using conveyor belts and transferred to a final rinse tank containing chlorine-free water to eliminate any residual disinfectant. It is essential to ensure that no disinfectant residues or pathogenic microorganisms are present in the final rinse water.

**Pre-drying**. The fruit undergoes a rapid pre-drying process in a hot-air tunnel, where excess water is removed.

**Sorting**. Fruits with visible deformities or rot are manually discarded. This is facilitated by passing the fruit through a tunnel illuminated with ultraviolet light, which aids in the detection of damage.

**Calibration**. An automatic calibration system classifies the fruits by size, discarding those that do not meet the calibration parameters.

**Drying**. Once the fruits have been washed and sorted for uniformity in size, weight, and colour, the fruit undergoes drying in a hot-air tunnel to ensure optimal adhesion of the subsequent coating.

**Preparation of the coating**. Commercial chitosan powder is dissolved in an acetic acid solution under continuous agitation at a temperature of 70 °C. The remaining components (glycerol and rosemary oil) are added one at a time, allowing each to dissolve in the mixture.

**Application of the coating**. The coating is applied using the “dipping” technique, which involves immersing the fruit in a tank containing the final coating solution for a specified duration to ensure thorough coverage of each piece.

**Draining**. The fruits are transferred to draining trays, where they are held for a predetermined period to remove excess coating.

**Coating drying**. The trays are then moved to a drying chamber at room temperature to facilitate coating drying.

**Packaging, labelling, and palletizing**. The oranges are packaged in plastic boxes with a capacity of 3 kg, labelled, and palletized.

**Storage**. The pallets are moved to a storage area where they are held for up to 12 weeks under controlled temperature and relative humidity conditions.

**Shipping**. The pallets are shipped in temperature-controlled refrigerated vehicles.

### 2.4. Food Defense

#### 2.4.1. Threat Identification

Food defense threats were identified through a structured assessment of the facility and processing operations using the Food Defense Self-Assessment Tool published by the U.S. Food and Drug Administration (FDA) [25]. The assessment was conducted by the research team through a systematic review of each stage included in the process flow diagram, from raw material reception to final shipping, in order to identify potential opportunities for intentional contamination or malicious interference.

Identified vulnerabilities were independently reviewed and discussed among the authors until consensus was reached regarding their inclusion in the final inventory of food defense threats. To reduce subjectivity, scoring decisions were discussed collectively by the authors and assigned only after agreement was reached regarding the characteristics and potential impact of each identified threat. This procedure resulted in the identification of 24 threats that were subsequently evaluated using the TACCP and CARVER+SHOCK methodologies.

Each identified threat was then assessed using two methods:

#### 2.4.2. TACCP Methodology

The significance of each identified threat was assessed using the TACCP methodology [26].

Significance was determined using the following formula:

(1) R = Likelihood × Impact

where

Likelihood: The probability of the event occurring.

Very high (5): Very high chance

High (4): High chance

Medium (3): Some chance.

Low (2): May happen.

Very low (1): Unlikely to happen.

Impact: The impact of the event if it occurs.

Very high (5): Catastrophic

High (4): Major

Medium (3): Significant

Low (2): Some

Very low (1): Minor

The TACCP assessment was based on the Likelihood × Impact approach proposed in PAS 96. For the purposes of this study, a simplified five-level risk classification system was adopted to facilitate interpretation of the results and comparison with the CARVER+SHOCK assessment. The adaptation did not modify the scoring procedure itself but only the categorization of the resulting scores into five qualitative risk levels (very low, low, moderate, high, and very high);

Very high (17–25)

High (10–16)

Moderate (5–9)

Low (3–4)

Very Low (1–2)

Although scores of 17–19 cannot be generated mathematically through multiplication of integer likelihood and impact values ranging from 1 to 5, these values were included within the “Very High” category to maintain a continuous and complete classification scale.

#### 2.4.3. CARVER+SHOCK Methodology

To improve consistency, each CARVER+SHOCK dimension was evaluated using predefined criteria adapted from published applications of the methodology [27]. CARVER+SHOCK evaluates risks across seven dimensions:

Criticality (C): The significance of the target in terms of operational goals or public health. Higher scores were assigned to targets whose intentional contamination could affect a larger quantity of product, compromise consumer health, or generate significant economic consequences.

Accessibility (A): The ease with which the target can be reached. Higher scores were assigned to locations that could be easily reached without special access permissions or security barriers.

Recoverability (R): The time and effort required to recover from an attack or failure. Higher scores indicated situations requiring longer recovery periods, greater operational disruption, or extensive corrective actions.

Vulnerability (V): The susceptibility of the target to disruption or compromise. Higher scores reflected targets that could be successfully contaminated with minimal effort or technical expertise.

Effect (E): The magnitude of impact caused by the attack or failure. Higher scores corresponded to events capable of producing greater public health, operational, economic, or reputational consequences.

Recognizability (R): The ease with which an adversary can identify the target as a critical point. Higher scores were assigned to targets that could be easily identified as critical process points by a potential attacker.

Shock (S): The psychological and economic impact of the event on stakeholders. Higher scores reflected situations likely to generate significant public concern, reputational damage, media attention, or economic impact.

Scores ranging from 1 to 10 were assigned to each dimension according to Table 1, based on the characteristics of the identified threat, facility layout, process design, accessibility conditions, and expected consequences.

Each factor was scored on a scale of 1 to 10, with higher scores indicating greater risk. The scoring process was conducted by the research team as part of the overall food defense assessment. Individual scores were assigned after a review of the process characteristics and identified vulnerabilities and were subsequently discussed among the authors until consensus was reached. This approach was adopted to improve consistency and reduce individual scoring bias. The scoring of each threat was based on a structured assessment of the facility characteristics, accessibility conditions, process layout, potential contamination opportunities, expected consequences, and recovery requirements. For each identified threat, all seven CARVER+SHOCK dimensions were evaluated independently before calculating the total score.

The total score was calculated as the sum of all dimensions and was used to prioritize threats for mitigation. High-risk threats (critical nodes) were defined as those with scores exceeding 50. TACCP and CARVER+SHOCK were selected because they represent two of the most widely recognized food defense assessment methodologies described in international guidance documents and the scientific literature. TACCP is strongly linked to PAS 96 recommendations and is commonly used in food defense programmes within the food industry due to its relatively simple and practical approach. In contrast, CARVER+SHOCK is a multidimensional vulnerability assessment methodology specifically designed to identify and prioritize targets susceptible to intentional contamination. The simultaneous application of both approaches enables a comparison of a semi-quantitative and a multidimensional assessment framework within the same industrial context.

## 3. Results and Discussion

### 3.1. Food Defense Threat Identification

Food defense threats were identified. Employee-related, visitor-related, contractor-related, and infrastructure-related vulnerabilities were included in the assessment through the application of the FDA Food Defense Self-Assessment Tool. Although insider threats are recognized as an important component of food defense programmes, the existing controls at the studied facility, including food defense training, visitor management procedures, restricted-access policies, and physical security measures, reduced the significance of these vulnerabilities relative to the process-related threats identified. Consequently, the most relevant vulnerabilities identified in the assessment were associated with specific processing operations and are presented in Table 2.

The food defense threats identified in the edible-coated orange processing line reflect a broader pattern observed in minimally processed fruit and vegetables (MPFV) operations. These products are often handled without a final microbial kill step, making them especially susceptible to intentional contamination. As noted in [28], MPFV are consumed raw and undergo only moderate microbial reduction during processing, which increases their risk profile for both food safety and food defense threats. These processes often involve open systems, manual handling, and multiple entry points for raw materials and additives, factors that collectively increase the risk of intentional contamination. The findings in Table 2 align with previous studies that emphasize the heightened vulnerability of fresh produce facilities due to their limited physical barriers and reliance on human oversight [4].

In particular, the reception phase presents multiple threats, including the potential adulteration of chemical additives and substitution of ingredients used in the coating formulation. These risks are exacerbated by the lack of verification mechanisms beyond documentation, a concern echoed in the work of Mitenius et al. [5], who argue that supplier assurance programmes often fail to detect intentional threats unless complemented by targeted risk assessments. In the case of oranges, the use of chemical additives such as imazalil and sodium hypochlorite introduces specific threats related to storage and dosing. Similar risks have been documented in leafy greens and fresh-cut vegetables, where chlorine-based disinfectants are widely used but often stored in unsecured areas, creating opportunities for tampering [29]. The lack of continuous monitoring of chemical parameters, as observed in this study, mirrors findings in salad processing facilities where pH and chlorine levels fluctuate due to inconsistent oversight.

The processing stages, such as washing, coating, and drying, are characterized by open equipment designs and limited access control. These features are common in citrus and other fruit processing lines, where hygiene and airflow requirements often conflict with physical security measures [15]. The dipping tanks and ambient drying chambers, for example, represent critical control points that are rarely addressed in conventional food defense plans. In this regard, previous work [30] and the FDA’s CARVER+SHOCK framework [11] highlight the importance of identifying such nodes where an attacker could cause maximum disruption with minimal effort. Furthermore, the manual sorting and grading phase introduces human factors that are difficult to control. As noted in [31], insider threats remain one of the most underestimated risks in food defense, particularly in facilities with high labour turnover or limited staff training. The lack of continuous monitoring during these stages increases the likelihood of intentional tampering going unnoticed.

The final stages—packing, labelling, and shipping—also pose relevant threats. Manipulation of labels and traceability data can compromise product integrity and regulatory compliance. This is consistent with findings from the Fresh Produce Safety Centre Australia & New Zealand [32], which identified traceability fraud as a growing concern in export-oriented fruit sectors. Specifically, these concerns are echoed in studies on strawberry tampering incidents in Australia, where fraudulent labelling and physical contamination led to widespread public alarm and economic losses. The parallels between citrus and other fruits suggest that threats are not product-specific but rather systemic across fresh produce operations.

Overall, threats identified throughout the edible-coated orange processing line are, in many respects, consistent with those typically observed in fresh produce operations. As noted in previous studies, processes involving minimally processed fruits often share common food defense threats, such as unsecured access to raw materials, open equipment designs, and limited surveillance during manual handling stages [28,32]. These threats are considered logical and expected in environments where hygiene and airflow requirements often limit the implementation of physical barriers [29]. It is important to note that most of the identified vulnerabilities are not unique to edible-coated orange production and are commonly encountered in conventional fresh-produce processing facilities. Examples include risks associated with raw material reception, chemical storage, washing systems, storage areas, transportation, and traceability controls.

The specific contribution of the edible coating process is the introduction of additional processing stages requiring independent food defense evaluation. These include the reception of coating ingredients, coating formulation and preparation, dipping operations, draining activities, drying chambers, and the storage of coating-related materials and utensils [15]. These additional steps create new vulnerability nodes that are not typically present in conventional citrus packing operations and therefore require dedicated assessment within food defense plans.

For example, the dipping tanks and ambient drying chambers used in the coating process represent new potential threat points in the process due to their open design and prolonged exposure times. These elements are not commonly found in traditional citrus packing lines and therefore require tailored risk assessment approaches. The combination of conventional and novel threats underscores the importance of conducting a detailed and context-specific risk analysis, which will be addressed in the following section.

Nevertheless, it should be acknowledged that insider threats can never be eliminated and may remain relevant even in facilities with established food defense programmes. Consequently, periodic reassessment of personnel-related vulnerabilities remains necessary as part of continuous food defense improvement.

### 3.2. Comparative Risk Assessment Outcomes

#### 3.2.1. Overall Comparison of TACCP and CARVER+SHOCK

Once the food defense threats were identified throughout the production process of whole oranges with edible coatings, a risk assessment of these threats was conducted using two distinct methodologies: TACCP and CARVER+SHOCK. The two methodologies differ substantially in their approach, scope, and results. This study evaluates both to provide a comprehensive perspective on their characteristics and practical usability. The contribution of this study should be interpreted as a comparative assessment of two established food defense methodologies within an emerging processing context rather than a validation of one methodology against another. The value of the comparison lies in understanding how different assessment frameworks influence threat prioritization, mitigation planning and resource allocation when applied to the same process. This perspective is particularly relevant for food companies and practitioners who must select a suitable vulnerability assessment methodology despite the absence of harmonized regulatory guidance regarding specific risk assessment tools.

The purpose of this comparison is not to validate one methodology against the other or to demonstrate methodological superiority. Instead, the study examines how different food defense assessment frameworks characterize and prioritize vulnerabilities when applied to the same processing environment. This comparative perspective provides practical information for food businesses facing the challenge of selecting an appropriate vulnerability assessment methodology in the absence of harmonized assessment criteria.

In terms of application to this process, TACCP is a straightforward, semi-quantitative method rooted in the principles of HACCP, designed to assess threats based on likelihood and impact. TACCP is particularly useful because it is easily integrable into existing food safety management systems. In fact, it is recommended by standards such as PAS 96 [26]. TACCP emphasizes strategic thinking and team-based evaluations, making it accessible to this type of middle-sized organization. In contrast, CARVER+SHOCK is a quantitative, multidimensional tool originally developed for military applications and later adapted for threat assessments in the pharmaceutical and food sectors [33,34]. This method evaluates seven attributes: Criticality, Accessibility, Recoverability, Vulnerability, Effect, Recognizability, and Shock, to score and rank potential targets for intentional contamination. This method allows for a more granular analysis and is especially valuable in high-risk environments or complex supply chains.

In the context of this orange-processing industry, application of the CARVER+SHOCK methodology to the production process of whole oranges with edible coatings proved to be significantly more complex than anticipated. This complexity stems from the method’s multidimensional nature, which requires the evaluation of seven distinct attributes, and each attribute requires specific, detailed input data, ranging from operational layouts and access controls to psychological and economic impact assessments. As a result, the assessment process required a broader and more granular collection of information than was required for the simpler semi-quantitative TACCP method. This observation aligns with findings from vulnerability assessments conducted in the meat processing industry, where practitioners reported that CARVER+SHOCK required extensive preparatory work and detailed facility-specific data to ensure accurate scoring [35]. The need for interdisciplinary collaboration and technical documentation further increases the resource intensity of the method, making it more suitable for organizations with robust data management systems and trained personnel. Despite these challenges, the depth of analysis provided by CARVER+SHOCK offers a more differentiated ranking of threats in the studied organization, which is particularly valuable in high-risk or complex food production environments such as the studied one.

For example, the open design of the washing tanks received high Accessibility and Vulnerability scores because the tanks were directly accessible during operation and could potentially allow the intentional addition of contaminants with minimal technical expertise. Likewise, high Criticality and Effect scores were assigned because contamination at this stage could affect a large volume of product and potentially compromise consumer health.

In contrast, threats such as visual inspection limited to obvious damage received lower CARVER+SHOCK scores because, although the vulnerability exists, the accessibility, effect, and shock dimensions were considered less severe within the context of the studied facility.

#### 3.2.2. Identification of Critical Vulnerabilities

Regarding risk level identification, the TACCP and CARVER+SHOCK assessments showed substantial agreement in identifying high-risk threats. In Table 3, both methods classified the lack of restricted access to Imazalil storage, the open design of washing tanks and the adulteration of additives during their manufacture or transport, and are identified as high-priority risks. Attacks on water supplies have been reported to be among the most frequent and easiest means of attacking the food chain. Several authors have described extortion attempts involving chemical agents in water supplies, including cyanide, pesticides, insecticides, herbicides, and rodenticides [36,37,38]. Biological agents have been reported as potential means of intentionally contaminating water supplies in the food chain [39]. In this study, both methods clearly identified the washing-tank vulnerability as a priority.

These results suggest that both methods are capable of identifying high-risk threats that could seriously compromise food safety. This capability has been observed in studies demonstrating the effectiveness of both methods for this purpose [7,8], which is a primary purpose of this type of analysis. The CARVER+SHOCK method has been successfully used to identify high-risk threats in catering companies [27] and in primary production [40], whereas TACCP has been successfully used for this purpose in dairy factories [41]. GFSI-recognized standards such as IFS [12] and BRCGS [2] require a procedure for identifying potential threats and a documented assessment of the associated risk. The present findings indicate that both methods, TACCP and CARVER+SHOCK, are fully capable of meeting these requirements because both methods identify high-risk food defense threats.

#### 3.2.3. Vulnerability Prioritization and Risk Differentiation

Regarding action-oriented recommendations, which should be an outcome of these risk analyses, both methods identify priority actions aimed at improving operational security. With respect to access control, inadequate control has been reported as a high-impact vulnerability in a food defense plan developed for military organizations [42]. It is therefore logical that both methods identified access control to storage as an area that requires immediate attention and action. However, because TACPP is a semi-quantitative method, it does not provide as differentiated a priority ranking as CARVER+SHOCK.

Regarding methodological complexity, TACCP uses a much simpler formula that combines likelihood and impact [26], producing a result that, although semi-quantitative, is valid for the intended purpose. For example, the absence of visual control during dosing operations, open conveyor belts facilitating contaminant introduction, and the inadequate access control store are all assigned scores corresponding to very high risk, indicating an equal and immediate need for mitigation measures for all these threats.

Nevertheless, CARVER+SHOCK applies a multidimensional scoring system across seven factors to produce a total score [34]. As shown in Table 3, the same threats (lack of visual control during dossing and open conveyor belts facilitating contaminant introduction) received scores of 48 and 43, respectively. Thus, although these threats are important, they are not classified as critical risks and do not rank above inadequate access control to store on the mitigation priority list, illustrating the broader strategic implications of this assessment method. This result may be explained by the recognizability factor in the CARVER+SHOCK, which is not directly considered in TACCP and contributes to the lower CARVER+SHOCK scores for these threats. Similar results were reported in a CARVER+SHOCK study of an onion production enterprise, in which recognizability was an important parameter in defining a lower risk indicator for the onion processing phase [40].

Regarding medium-rated risks, the methods agreed on most of the threats. In TACCP, threats such as visual inspection limited to obvious damage and manual handling without monitoring are classified as moderate risk (scores of 9 and 8, respectively), whereas CARVER+SHOCK assigns scores of 42 and 40, respectively, indicating non-critical risk while expressing their criticality and potential impact more specifically. In terms of scope and perspective, TACCP focuses on immediate operational risks, making it suitable for daily monitoring and routine evaluations, whereas CARVER+SHOCK adopts a strategic approach that considers long-term consequences, economic impacts, and psychological factors [43]. For example, the adulteration of Imazalil receives a critical score of 55, indicating severe and long-term implications beyond the production line. In terms of psychological and economic dimensions, CARVER+SHOCK uniquely incorporates the “Shock” factor [27], assessing the social and economic repercussions of a vulnerability. This dimension is absent in TACCP, which focuses primarily on operational impacts [26].

The most important difference between these methodologies lies in their approach to vulnerability prioritization. TACCP categorizes threats into relatively broad risk levels, whereas CARVER+SHOCK generates a more differentiated ranking through its multidimensional scoring framework. However, this greater differentiation should not be interpreted as evidence of superior accuracy or validity, but rather as a different approach to supporting decision-making and resource allocation.

#### 3.2.4. Implications for Mitigation Planning

Threats such as the open design of washing tanks, Imazalil storage without restricted access and unrestricted access to dipping tanks are classified as very high risk by TACCP, which assigns the same level of importance to all of them. In contrast, CARVER+SHOCK assigns these specific scores of 56, 55 and 53, respectively, because this method considers potential social and economic repercussions. These quantitative factors enable a more precise prioritization of the mitigation plan. Several authors have demonstrated that this quantitative method can be applied successfully to develop coherent, prioritized mitigation plans in settings such as catering and primary production [27,40].

Although CARVER+SHOCK generated a wider distribution of scores and a more differentiated ranking of threats, this characteristic reflects the greater scoring resolution inherent to its multidimensional assessment structure. In this way, organizations may be able to allocate their mitigation budget by prioritizing what truly requires immediate attention. Nevertheless, greater scoring resolution should not be interpreted as evidence of greater accuracy or validity in the absence of independent validation procedures.

TACCP is a simpler and faster method that, when applied appropriately, is valid for determining significance in a straightforward manner. The differences between these approaches highlight their respective strengths for the overall vulnerability analysis. TACCP is suitable for organizations seeking a clear and practical framework for managing day-to-day operational risks [41]. Its simplicity allows for quick evaluations and efficient resource allocation. CARVER+SHOCK is invaluable for strategic risk management, particularly in industries where threats can have far-reaching consequences. By considering factors such as recoverability and psychological impact, CARVER+SHOCK enables more informed decision-making for long-term vulnerability mitigation. An advantage of TACCP is that it does not underestimate significant risks; rather, it tends to overestimate them. Therefore, this method can be used for rapid detection when there is no pressing need to fine-tune budgets by prioritizing mitigation strategies.

Considering the results obtained throughout this study and the comparative analysis of both methodologies, the CARVER+SHOCK method generated a more differentiated prioritization of vulnerabilities within the studied facility due to its multidimensional scoring framework. Despite requiring a larger volume of data input and specialized training for evaluators, it generated a higher degree of ranking resolution among identified threats because it considers multiple assessment dimensions. This characteristic may be beneficial for organizations seeking a more detailed ranking of identified threats.

These results also enable the company to allocate its budget more effectively by prioritizing mitigation measures that are critical to the specific process under evaluation.

It is important to note that the greater numerical differentiation provided by CARVER+SHOCK is an inherent consequence of its multidimensional scoring framework and broader scoring scale. Therefore, a greater spread of scores should not be interpreted as evidence of superior accuracy, validity, or effectiveness. Rather, it reflects a higher level of ranking resolution, which may facilitate the prioritization of vulnerabilities within the specific context evaluated.

In addition, it should be noted that the present study does not include independent validation of the assigned risk scores, comparison with historical food defense incidents, inter-rater reliability analysis, or post-implementation evaluation of mitigation effectiveness. Consequently, the results do not allow conclusions regarding the relative accuracy, validity, or predictive performance of TACCP and CARVER+SHOCK. The comparison should therefore be interpreted as an evaluation of how the two methodologies prioritize vulnerabilities within the specific context of the studied facility.

Both methodologies demonstrated the ability to identify vulnerabilities requiring mitigation measures within the studied process. TACCP provides a simpler and more straightforward assessment framework, whereas CARVER+SHOCK generates a more differentiated ranking structure. The selection of one methodology over another may therefore depend on organizational objectives, available resources, and the desired level of detail in vulnerability prioritization.

To facilitate interpretation of the comparative findings, Table 4 summarizes the main characteristics, strengths, limitations, and potential application contexts of TACCP and CARVER+SHOCK based on the observations obtained in the present case study.

### 3.3. Food Defense Mitigation Strategy

In the context of food defense, a mitigation plan consists of strategies that protect vulnerable facilities and processes against potential malicious attacks [44]. As reported by Navarrete et al. [45], the plan should be established according to the priorities established by the risk assessment. It should also specify the actions, resources and responsibilities. Once implemented, the plan must be verified to ensure its effectiveness [44].

The mitigation plan was developed as a hypothetical implementation scenario based on the relative prioritization of vulnerabilities obtained through the TACCP and CARVER+SHOCK assessments. The proposed implementation sequence was intended to illustrate how vulnerability assessment results may support the planning of mitigation measures within an industrial food defense programme.

Estimated implementation deadlines and investment ranges were derived from expert judgement, considering the expected complexity of each mitigation measure, operational requirements, infrastructure modifications, equipment needs, personnel involvement, and available information from the studied facility. These estimates should be interpreted as illustrative planning values rather than validated economic assessments or actual implementation records. The mitigation plan developed in this study is shown in Table 5.

As shown in Table 5, threats involving water use, such as those at the pre-washing and washing stages, are among the highest priorities for mitigation because water is considered one of the easiest routes for a malicious attack. Khan et al. (2001) [22] reported several preventive measures against water sabotage, including routine checks for chemical or microbiological tampering. This company has implemented a water control plan to ensure food safety according to Royal Decree 3/2023 [46]. Routine monitoring should be performed weekly and should include (i) colour, taste, and odour control; (ii) turbidity; (iii) pH; and (iv) residual free chlorine. Given the high risk associated with this threat, the company should consider conducting this check daily. In addition, these stages involve open tanks in which an intentional act of contamination could be readily committed. Praia & Henriques (2021) [47] proposed video surveillance as an effective means for enhancing security and reducing intentional food adulteration incidents. Therefore, installing security cameras in these areas should be considered as a means of mitigating sabotage risk. The same measure could also be applied in other areas or stages, including open conveyor belts; stages involving direct employee handling, such as sorting, grading, and coating preparation; and areas in which the product comes into contact with media other than water, such as the air tunnel. However, installing security cameras may require substantial investment; therefore, priority should be given to the areas at greatest risk of malicious attack, as shown in Table 3: (i) pre-washing, (ii) washing, (iii) coating-film preparation, and (iv) packing, labelling, and palletizing.

A food business should prioritize restricting access to potential targets in its food defense strategy because this is an aspect it can manage effectively. Therefore, access to critical areas, such as drenching dosage, application, draining, and drying of the film, and storage, and the access to chemical products, such as imazalil and sodium hypochlorite storage, must be controlled. Rasco and Bledsoe [48] reported the effectiveness of access control by locking with access only to authorized personnel.

Regarding threats associated with the reception of raw materials, several mitigation measures may be considered, as previously described by Andrade et al. [49]. First, supplier audits are useful for mitigating threats. Second-party audits assess supplier performance through inspection of the manufacturing process [50], thereby reducing the risk of product contamination, including intentional contamination. Another option proposed by Kotsanopoulos and Arvanitoyannis [50] is to request certification to a GFSI-recognized standard [1,2]. Such certification gives great confidence to suppliers because of the stringent requirements of these standards, including food defense requirements. In addition to supplier audits, another effective measure includes monitoring the vehicles used in the transport, conducting reception-stage controls to ensure that raw materials come from authorized suppliers, and inspecting packaging integrity. Finally, controlling raw material traceability is another essential measure for mitigating the threats at the reception stage [49].

For Imazalil and sodium hypochlorite dosage, the associated parameters must be controlled because of the high chemical threat posed by these compounds. Imazalil is used to control fungal growth in oranges, but incorrect dosing may harm consumers’ health because the substance may affect the liver [23]. Sodium hypochlorite can cause substantial toxicity if ingested in large amounts, including injuries to the gastrointestinal tract and respiratory problems, among others [24]. Ideally, monitoring systems should operate to detect deviations promptly [51]. Moreover, in the context of food defense strategies, the monitoring equipment should include an alarm system that provides a warning when any monitored parameter deviates from its specified range.

Regarding the poor transportation security identified at the shipping stage, Jurica et al. [8] considered this threat to pose a high risk for malicious attacks. They therefore proposed the following mitigation measures: locking the vehicle’s cargo area, providing advance notice of delivery, and recording the employee’s name and the vehicle’s registration mark.

Finally, traceability must also be controlled. Puhač Bogadi et al. [52] considered the digitalization of traceability to be a risk factor because of the frequency of current cyberattacks. Accordingly, these authors proposed a blockchain system as a mitigation measure against the potential tampering of traceability data. Among all the benefits of blockchain, data immutability stands out. Once information is recorded on the chain, it cannot be modified without leaving a trace, thereby reducing the risk of fraud or data manipulation [53].

According to the results obtained from the vulnerability assessment using both methods, a total of 24 threats were identified, so the company should implement mitigation strategies to address them. However, the economic investment that the company would have to make to address all these measures is high, as shown in Table 5. Therefore, the company may be unable to incur the entire expense at once. For this reason, it is imperative to clearly identify risks and establish realistic timelines according to the identified priorities.

The TACCP results in this case study included only four risk levels (very high, high, moderate, and low). Therefore, when defining the timeframes for implementing control measures, the company will establish a deadline for each of these levels: (i) 1 month for very high-risk threats; (ii) 4 months for high-risk threats; (iii) 8 months for moderate risk threats, and (iv) 12 months for low risk (Table 5). However, it is worth noting that each risk level comprises many mitigation measures. For instance, to mitigate all threats defined as very high risk, a total of 12 control measures should be implemented. However, TACCP does not provide any criteria for deciding which of these threats should be addressed first at each level. This could hinder the company’s decision-making process.

This issue is less evident with CARVER+SHOCK, which establishes a clear priority order (Table 3) and allows more specific timeframes to be assigned to each mitigation measure. The company can therefore establish an action schedule based on the priority order obtained in the vulnerability assessment (Table 5). In this way, the company has a clearly defined roadmap for implementing the necessary mitigation measures according to risk. The CARVER+SHOCK prioritization exercise, for example, suggests that installing security cameras in the pre-washing and washing areas should be among the earliest mitigation actions because both threats received the highest score of 56. As shown in Table 5, a one-month implementation target was assigned to these measures for illustrative planning purposes. Access controls at the drenching-dosage stage could then be implemented within 1.5 months, following their priority score of 55, while access controls for coating application, draining, drying, and storage could be implemented within 2 months, following their priority score of 53. This approach would be applied to each mitigation strategy in the plan. This priority order is less clear when TACCP is used. Based on the above, CARVER+SHOCK appears to provide a clearer approach than TACCP for designing and implementing a mitigation plan within a food defense plan. Nevertheless, the mitigation schedule presented in Table 5 should be interpreted as a planning example derived from the vulnerability assessment results. Actual implementation priorities, costs, and timelines will depend on the specific economic, operational, and organizational conditions of each facility. Although the vulnerability rankings presented in this study are specific to the evaluated facility, the overall assessment framework may be transferable to other fresh-produce industries and minimally processed food systems. Many of the vulnerabilities identified, including those associated with open processing equipment, water-based operations, ingredient handling, storage areas, transportation logistics, and traceability systems, are not unique to citrus processing and may also be relevant in the production of fresh-cut fruits, minimally processed vegetables, berries, table grapes, and other fresh produce products.

Furthermore, the additional vulnerability nodes introduced by edible coating technology share characteristics with other emerging postharvest technologies that incorporate supplementary ingredients, formulation stages, dipping systems, or extended product handling operations. Consequently, the comparative application of TACCP and CARVER+SHOCK may provide a useful framework for evaluating food defense vulnerabilities in a broader range of minimally processed food systems. Nevertheless, the specific vulnerabilities identified, their relative prioritization, and the resulting mitigation strategies should always be adapted to the operational, organizational, and infrastructural characteristics of each facility.

### 3.4. Study Limitations

The findings of this study should be interpreted in light of several limitations. First, the assessment was conducted within a single industrial facility, which may limit the direct transferability of the results to other citrus-processing operations, food sectors, or edible-coating systems. Differences in facility design, production scale, organizational practices, security controls, and process configurations may influence vulnerability identification and prioritization outcomes.

Second, the assessment relied on expert judgement and consensus among the authors, and no independent validation, inter-rater reliability analysis, or post-implementation evaluation was performed. Consequently, the study should be interpreted as a comparative case study of vulnerability prioritization outcomes rather than a validation of either methodology.

Future research should include additional case studies across different processing environments and food sectors, as well as independent validation approaches, to further evaluate the robustness and generalizability of the observed findings.

## 4. Conclusions

This study provides a comprehensive food defense vulnerability assessment of a fresh orange processing line incorporating edible coating technology, an emerging postharvest innovation that introduces additional complexity and previously underexplored intentional contamination risks. By applying and comparing two widely recognized methodologies, TACCP and CARVER+SHOCK, this study demonstrates that both approaches are capable of identifying critical food defense threats and meeting the requirements of GFSI-recognized standards and the U.S. FSMA IA Rule. Both approaches consistently highlighted high-risk vulnerabilities associated with water-based operations, chemical storage and dosing, open processing equipment, and inadequate access controls in coating-application and storage areas.

However, significant differences were observed in the prioritization and practical usability of the two methods. TACCP proved to be a simple, intuitive, and rapidly deployable tool that can be easily integrated into existing food safety management systems. Its tendency to overestimate risks ensures a conservative approach that minimizes the likelihood of underestimating critical threats. Nevertheless, its semi-quantitative nature limits its ability to differentiate priorities among high-risk threats, which may complicate decision-making when resources are constrained. In contrast, CARVER+SHOCK offered a more detailed and quantitative prioritization of vulnerabilities by incorporating strategic, economic, and psychological impact dimensions. Although its application requires greater data availability, technical expertise, and time investment, this method enabled a clearer hierarchy of mitigation actions and more efficient allocation of resources. For the studied medium-sized citrus processing facility, CARVER+SHOCK provided a more differentiated prioritization of the identified threats, which facilitated the design of a structured and prioritized food defense mitigation plan.

From a scientific perspective, this study contributes to the limited literature comparing food defense assessment methodologies within emerging fresh-produce processing systems. Rather than demonstrating the superiority of one methodology over another, the results illustrate how different assessment frameworks may lead to different threat prioritization outcomes and therefore influence the design and implementation of food defense mitigation strategies.

From a practical perspective, both methodologies proved capable of identifying vulnerabilities requiring mitigation within the evaluated facility. TACCP provided a simple and easily deployable framework that can be readily integrated into existing food safety management systems, while CARVER+SHOCK generated a more differentiated prioritization of identified vulnerabilities through its multidimensional assessment structure. These characteristics may support food businesses when selecting a vulnerability assessment approach according to their operational objectives, available resources, and desired level of assessment detail.

The results presented in this study are specific to the evaluated facility and should not be interpreted as representative of the entire citrus-processing sector or edible-coating industry. Future research involving multiple facilities, different process configurations, and independent validation approaches will be necessary to assess the broader applicability and robustness of the findings reported here.

## Figures and Tables

**Table 1:** Scoring guidance used during the CARVER+SHOCK assessment.

Dimension	Score 1–3	Score 4–6	Score 7–10
Criticality	Limited impact	Moderate impact	Severe health/economic impact
Accessibility	Restricted access	Controlled access	Open access
Recoverability	Rapid recovery	Moderate recovery	Difficult recovery
Vulnerability	Difficult attack	Moderate difficulty	Easy attack
Effect	Limited consequences	Moderate consequences	Severe consequences
Recognizability	Difficult to identify	Moderately recognizable	Easily recognizable
Shock	Limited public concern	Moderate concern	Major public/economic concern

**Table 2:** Food defense threats in an edible-coated oranges processing plant.

Phase	Food Defense Risks
Reception of raw materials (oranges, additives, ingredients, packaging materials).	*Oranges:* Visual inspection is limited to obvious damage to the boxes and does not assess possible intentional contamination. Risk posed by unreliable suppliers or by suppliers that are not audited periodically beyond GLOBAL G.A.P. certification. *Additives (imazalil, sodium hypochlorite):* Possible adulteration of chemical products during manufacturing or transportation if the containers are not secure or sealed. Lack of verification, beyond documentation review, that the content meets the specifications in the analysis report. Ingredients for the coating film *(chitosan, acetic acid, rosemary extract, glycerol):* Risk of contamination if containers are stored without access controls. Risk of adulteration or substitution with lower-quality products if suppliers do not strictly meet specifications. *Packaging and labelling materials:* Labels and boxes could be tampered with, resulting in fraudulent information or compromising traceability. Wooden pallets could conceal contaminants or facilitate unauthorized personnel access.
Drenching dosage (Imazalil)	Lack of continuous visual control in the area where the fungicide is dosed, which could allow the intentional addition of harmful substances. Failure to enforce restricted, monitored storage of imazalil could create a vulnerability.
Dumping and pre-washing	Recirculating water can be a vector for intentional contamination if access to and quality of the water are not strictly controlled. The open design of conveyor belts can facilitate the introduction of chemical contaminants during transportation.
Washing	If sodium hypochlorite is not stored securely, it may be tampered with. A lack of continuous supervision of pH and chlorine parameters could reduce process effectiveness and create opportunities for subsequent contamination. The open design of washing tanks can facilitate the introduction of chemical contaminants during washing.
Pre-drying	Risk of contaminant introduction if the hot air tunnel does not have strict access controls and regular maintenance.
Sorting and grading	Manual handling during sorting exposes the fruit to a higher risk of intentional tampering, especially if operator monitoring is insufficient.
Coating film preparation	If containers or utensils are not secured, they could be tampered with.
Coating application, draining, and drying	The dipping tank could be a critical contamination point if access is not restricted or if it is not inspected before processing. The ambient temperature drying chamber can be a risk area if access is not controlled during the prolonged drying time (2 h).
Packing, labelling, and palletizing	Manipulation of labels to change traceability information or adulterate product data. Intentional contamination during the palletizing process if areas are not properly monitored.
Storage and shipping	During prolonged storage, uncontrolled access to the area could allow product adulteration. Transportation in temperature-controlled vehicles can be a vulnerable point if the security of access doors and continuous load monitoring are not ensured.

**Table 3:** Risk assessment of food defense threats using TACCP and CARVER+SHOCK.

**Reception of Raw Materials (Oranges, Additives, Ingredients, Packaging Materials)**											
Threat	L	I	R1	C	A	R	V	E	R	S	**R2**
Visual inspection limited to obvious damage	3	3	9 (Moderate)	6	6	7	6	6	6	5	**42**
Unreliable suppliers/not regularly audited	1	3	3 (Low)	7	5	5	5	6	6	5	**39**
Adulteration of additives during manufacturing or transportation	4	5	20 (Very high)	8	7	6	7	8	7	7	**50**
Lack of additional verification of additive specifications	2	3	6 (Moderate)	6	6	7	6	7	6	5	**43**
Contamination of ingredient containers due to lack of access control	3	5	15 (High)	8	7	5	7	8	7	7	**49**
Adulteration or substitution by ingredient suppliers	1	5	5 (Moderate)	6	5	7	6	7	5	5	**41**
Tampering with labels or boxes compromising traceability	3	5	15 (High)	7	7	6	6	8	6	7	**47**
Wooden pallets facilitating unauthorized access	1	3	3 (Low)	6	6	6	5	6	6	5	**40**
**Drenching dosage (Imazalil)**											
Threat	L	I	R1	C	A	R	V	E	R	S	**R2**
Lack of continuous visual control during dosing	4	5	20 (Very high)	8	7	6	7	8	5	7	**48**
Imazalil storage without restricted access	4	5	20 (Very high)	9	8	6	8	9	7	8	**55**
**Dumping and pre-washing**											
Threat	L	I	R1	C	A	R	V	E	R	S	**R2**
Open design of recirculating water tank	4	5	20 (Very high)	9	9	6	8	9	7	8	**56**
Open conveyor belts facilitating contaminant introduction	4	5	20 (Very high)	7	6	6	6	7	5	6	**43**
**Washing** (**with sodium hypochlorite**)											
Threat	L	I	R1	C	A	R	V	E	R	S	**R2**
Unsafe storage of sodium hypochlorite	4	5	20 (Very high)	9	7	5	7	9	7	8	**52**
Lack of continuous supervision of critical parameters	3	5	15 (High)	8	7	6	6	8	6	7	**48**
Open design of washing tanks	4	5	20 (Very high)	9	9	6	8	9	7	8	**56**
**Pre-drying**											
Threat	L	I	R1	C	A	R	V	E	R	S	**R2**
Introduction of contaminants due to lack of control in the air tunnel	3	5	15 (High)	8	6	6	6	8	6	7	**47**
**Sorting and grading**											
Threat	L	I	R1	C	A	R	V	E	R	S	**R2**
Manual handling without monitoring	2	4	8 (Moderate)	6	5	5	6	6	6	6	**40**
**Coating film preparation**											
Threat	L	I	R1	C	A	R	V	E	R	S	**R2**
Tampering with unsecured containers or utensils	4	5	20 (Very high)	8	7	6	7	8	7	7	**50**
**Application, draining, and drying of the film**											
Threat	L	I	R1	C	A	R	V	E	R	S	**R2**
Dipping tank without access restrictions	4	5	20 (Very high)	9	7	6	7	9	7	8	**53**
Drying chamber without monitoring during prolonged drying	3	5	15 (High)	8	6	6	6	8	6	7	**47**
**Packing, labelling, and palletizing**											
Threat	L	I	R1	C	A	R	V	E	R	S	**R2**
Label tampering affecting traceability	3	5	15 (High)	7	7	6	6	8	6	7	**47**
Contamination during palletizing due to lack of monitoring	4	5	20 (Very high)	7	7	7	7	8	7	7	**50**
**Storage and shipping**											
Threat	L	I	R1	C	A	R	V	E	R	S	**R2**
Uncontrolled access during prolonged storage	4	5	20 (Very high)	9	7	6	7	9	7	8	**53**
Poor transportation security	4	5	20 (Very high)	8	7	6	7	8	7	7	**50**

L = Likelihood; I = Impact; R1 = Total risk TACCP; C = Criticality; A = Accessibility; R = Recoverability; V = Vulnerability; E = Effect; R = Recognizability; S = Shock; R2 = Total CARVER+SHOCK risk.

**Table 4:** Comparative characteristics of TACCP and CARVER+SHOCK.

Aspect	TACCP	CARVER+SHOCK
Assessment approach	Semi-quantitative (Likelihood × Impact)	Multidimensional vulnerability assessment
Main objective	Identification and categorization of threats	Detailed prioritization of vulnerabilities
Scoring structure	5 × 5 risk matrix	Seven dimensions scored from 1 to 10
Ease of application	High	Moderate to low
Data requirements	Relatively limited	More extensive
Training requirements	Basic food defense knowledge	Greater familiarity with CARVER+SHOCK criteria
Time required for assessment	Short	Longer
Strengths	Simple, intuitive, easy to communicate, readily integrated into food safety management systems	Provides a more differentiated ranking of identified vulnerabilities and considers multiple dimensions of impact
Limitations	Broad risk categories may reduce differentiation between high-priority threats	Increased complexity, greater dependence on detailed information and expert judgement
Recommended application	Routine assessments, SMEs, rapid screening exercises	Detailed vulnerability prioritization and strategic food defense planning
Output generated	Qualitative risk categories	Detailed vulnerability ranking

**Table 5:** Mitigation plan resulting from the TACCP and CARVER+SHOCK risk assessments.

Stage	Threat	Mitigation Measure	Priority Order		Estimated Implantation Period *		Estimated Investment Ranges *	
			TACCP	CARVER+SHOCK	TACCP	CARVER+SHOCK	TACCP	CARVER+SHOCK
Pre-washing	Open design of recirculating water tank	Installation of security cameras Routine checks for tampering	Very high	56	1 month	1 month	Medium	Medium
Washing	Open design of washing tanks	Installation of security cameras Routine checks for tampering	Very high	56	1 month	1 month	Medium	Medium
Drenching dosage	Imazalil storage without restricted access	Control access (locked area with access only to authorized personnel)	Very high	55	1 month	1.5 months	Low	Low
Application, draining, and drying of the film	Dipping tank without access restrictions	Control access (locked area with access only to authorized personnel)	Very high	53	1 month	2 months	Low	Low
Storage	Uncontrolled access during prolonged storage	Control access (locked area with access only to authorized personnel)	Very high	53	1 month	2 months	Low	Low
Washing	Unsafe storage of sodium hypochlorite	Control access (locked area with access only to authorized personnel)	Very high	52	1 month	3 months	Low	Low
Reception of raw materials	Adulteration of additives during manufacturing or transportation	Supplier audit. Control in the reception stage	Very high	50	1 month	3.5 months	Low	Low
Coating film preparation	Tampering with unsecured containers or utensils	Installation of security cameras	Very high	50	1 month	3.5 months	Medium	Medium
Packing, labelling, and palletizing	Contamination during palletizing due to lack of monitoring	Installation of security cameras	Very high	50	1 month	3.5 months	Medium	Medium
Shipping	Poor transportation security	Transport control (including locking up the transportation storage part, delivery announcement in advance and the registration of the name of the employee and the mark for the vehicle).	Very high	50	1 month	3.5 months	Low	Low
Reception of raw materials	Contamination of ingredient containers due to lack of access control	Control in the reception stage (sealed containers) Operators adequately trained in food defense (internal training).	High	49	4 months	4 months	Low	Low
Drenching dosage (Imazalil)	Lack of continuous visual control during dosing	Dosage control with alarm equipment	Very high	48	1 month	5 months	Low	Low
Washing (with sodium hypochlorite)	Lack of continuous supervision of critical parameters	Control of pH and free chlorine concentration with alarm equipment	High	48	4 months	5 months	Low	Low
Reception of raw materials	Tampering with labels or boxes compromising traceability	Control in the reception stage and blockchain system Operators adequately trained in food defense (internal training).	High	47	4 months	6–7 months	Low	Low
Pre-drying	Introduction of contaminants due to lack of control in the air tunnel	Installation of security cameras	High	47	4 months	6–7 months	Medium	Medium
Application, draining, and drying of the film	Drying chamber without prolonged control	Control access (locked area with access only to authorized personnel)	High	47	4 months	6–7 months	Low	Low
Packing, labelling, and palletizing	Label tampering affecting traceability	Blockchain system	High	47	4 months	6–7 months	High	High
Reception of raw materials	Lack of additional verification of additive specifications	Supplier audit. Control in the reception stage	Moderate	43	8 months	8 months	Low	Low
Dumping and pre-washing	Open conveyor belts facilitating contaminant introduction	Installation of security cameras Routine checks for tampering	Very high	43	1 month	8 months	Medium	Medium
Reception of raw materials	Visual inspection limited to obvious damage	Control in the reception stage (inspecting the packaging to verify its integrity) Operators adequately trained in food defense (internal training).	Moderate	42	8 months	9 months	Low	Low
Reception of raw materials	Adulteration or substitution by ingredient suppliers	Supplier audit. Control in the reception stage	Moderate	41	8 months	10 months	Low (audits) Low(operators training)	Low(audits) Low (operator training)
Reception of raw materials	Wooden pallets facilitating unauthorized access	Control access (locked area with access only to authorized personnel)	Low	40	12 months	11 months	Low	Low
Sorting and grading	Manual handling without monitoring	Installation of security cameras	Moderate	40	8 months	11 months	Medium	Medium
Reception of raw materials	Unreliable suppliers/not regularly audited	Supplier audit. Control in the reception stage	Low	39	12 months	12 months	Low (audits) Low (operator training)	Low (audits) Low (operator training)

* Implementation deadlines and estimated investment range (Low: limited investment required, mainly procedural or training measures, Medium: moderate investment associated with equipment, monitoring, or operational modifications, High: substantial investment involving infrastructure upgrades, security systems, or major process modifications) are illustrative values developed for this specific case study. They are based on expert judgement and available facility-specific information under its particular operating conditions. They do not represent actual implementation costs or validated economic analyses. The resulting categories (Low, Medium, and High investment) are intended to provide a relative indication of implementation effort rather than a formal economic assessment.

## Data Availability

The original contributions presented in this study are included in the article. Further inquiries can be directed to the corresponding author.
